# circ-CBFB upregulates p66Shc to perturb mitochondrial dynamics in APAP-induced liver injury

**DOI:** 10.1038/s41419-020-03160-y

**Published:** 2020-11-06

**Authors:** Zhecheng Wang, Yan Zhao, Ruimin Sun, Yu Sun, Deshun Liu, Musen Lin, Zhao Chen, Junjun Zhou, Li Lv, Xiaofeng Tian, Jihong Yao

**Affiliations:** 1 Department of Pharmacology, Dalian Medical University, 116044 Dalian, China; 2 Department of General Surgery, The Second Affiliated Hospital of Dalian Medical University, 116023 Dalian, China

**Keywords:** Cell biology, Molecular biology

## Abstract

p66Shc, a master regulator of mitochondrial reactive oxygen species (mtROS), is a crucial mediator of hepatocyte oxidative stress. However, its functional contribution to acetaminophen (APAP)-induced liver injury and the mechanism by which it is modulated remain unknown. Here, we aimed to assess the effect of p66Shc on APAP-induced liver injury and to evaluate if circular RNA (circRNA) functions as a competitive endogenous RNA (ceRNA) to mediate p66Shc in APAP-induced liver injury. p66Shc-, miR-185-5p-, and circ-CBFB-silenced mice were injected with APAP. AML12 cells were transfected with p66Shc, miR-185-5p, and circ-CBFB silencing or overexpression plasmids or siRNAs prior to APAP stimulation. p66Shc was upregulated in liver tissues in response to APAP, and p66Shc silencing in vivo protected mice from APAP-induced mitochondrial dynamics perturbation and liver injury. p66Shc knockdown in vitro attenuated mitochondrial dynamics and APAP-induced hepatocyte injury. Mechanically, p66Shc perturbs mitochondrial dynamics partially by inhibiting OMA1 ubiquitination. miR-185-5p, which directly suppressed p66Shc translation, was identified by microarray and bioinformatics analyses, and its overexpression attenuated mitochondrial dynamics and hepatocyte injury in vitro. Furthermore, luciferase, pull-down and RNA immunoprecipitation assays demonstrated that circ-CBFB acts as a miRNA sponge of miR-185-5p to mediate p66Shc in APAP-induced liver injury. circ-CBFB knockdown also alleviated APAP-induced mitochondrial dynamics perturbation and hepatocyte injury. More importantly, we found that the protective effects of circ-CBFB knockdown on p66Shc, mitochondrial dynamics and liver injury were abolished by miR-185-5p inhibition both in vivo and in vitro. In conclusion, p66Shc is a key regulator of APAP-induced liver injury that acts by triggering mitochondrial dynamics perturbation. circ-CBFB functions as a ceRNA to regulate p66Shc during APAP-induced liver injury, which may provide a potential therapeutic target.

## Introduction

Acetaminophen (APAP) is a safe antipyretic and analgesic drug at therapeutic levels. However, APAP overdose is the most common cause of acute liver failure in the US and UK^1^. APAP is metabolized in hepatocytes and converted by cytochrome P-450 2E1 (CYP2E1) to *N*-acetyl-p-benzoquinoneimine (NAPQI), which covalently binds to intracellular proteins, especially mitochondrial proteins, to form APAP adducts^2,3^. The formation of APAP adducts in the mitochondria results in mitochondrial dysfunction, releasing mitochondrial cell death factors and leading to nuclear DNA fragmentation and hepatocyte death^4–6^. Thus, hepatocyte survival and the therapeutic outcome of APAP-induced liver injury may benefit from treatments that minimize mitochondrial damage and/or enhance mitochondrial function.

Mitochondria are highly dynamic organelles that undergo remodeling to meet the metabolic demands of cells^7^. Mitochondrial dynamics, which is a general term that mainly includes mitochondrial fusion and mitochondrial fission, maintains healthy mitochondria within cells^8,9^. OMA1, which is an ATP-independent protease with activities overlapping with the m-AAA proteases, is identified as an essential factor of mitochondrial dynamics^10^. OMA1 overexpression triggers mitochondrial fission by cleaving L-OPA1 into S-OPA1^11^. In light of the recent study, mitochondrial reactive oxygen species (mtROS) play a major role in the OMA1-induced cleavage of OPA1^12^. However, the source and regulation of mtROS, as well as the specific mechanism of mtROS on OMA1, remain to be clarified.

P66Shc, which is an adapter protein that belongs to the ShcA protein family, has been regarded as a master regulator of mtROS in mammals and is thereby involved in a wide array of diseases^13,14^. Upon stress stimuli, p66Shc is activated and phosphorylated in the cytosol, and it then translocates to the intermembranous mitochondrial space, where it binds to and oxidizes cytochrome C to generate excessive mtROS^15,16^. Our previous work showed that p66Shc can serve as a crucial mediator and a therapeutic target in hepatocyte oxidative stress^17–19^. Furthermore, it was recently reported that p66Shc participates in some pathological processes by mediating mitochondrial dysfunction^20–23^. However, the potential role of p66Shc in APAP-induced liver injury remains unknown.

Circular RNAs (circRNAs), which are a novel class of noncoding RNAs, are highly conserved and are characterized as covalently closed-loop structures with neither a polyadenylated tail nor 5′-to-3′ polarity^24,25^. CircRNAs are produced by reverse splicing and are more stable than linear splicing products because of their resistance to exonucleases^26^. Moreover, circRNAs can be used as biomarkers for disease diagnosis^27,28^. Functionally, circRNAs can regulate gene expression through different mechanisms, including adsorbing microRNAs (miRNAs)^29–31^, binding to RNA-binding proteins^32^, and translating into peptides^33–35^. To date, the circRNAs that function as competing endogenous RNA (ceRNA) have been most extensively studied in various types of liver diseases. For example, circTRIM33-12 functions as an miRNA sponge to alleviate the progression of hepatocellular carcinoma^36^. Although the association of noncoding RNAs with liver diseases has been gradually recognized, the regulatory roles of circRNAs in APAP-induced liver injury as well as p66Shc are far from being understood.

The aims of this study were as follows: (1) to determine the potential role of p66Shc in APAP-induced liver injury; (2) to elucidate the underlying mechanisms by which p66Shc participates in APAP-induced liver injury by regulating mitochondrial dynamics; and (3) to test whether circRNA functions as a ceRNA to perturb mitochondrial dynamics and promote APAP-induced liver injury progression by upregulating p66Shc.

## Materials and methods

### APAP-induced liver injury in vivo

Adult male C57BL/6 mice (aged 8 weeks) weighing 20 ± 2 g were obtained from the Laboratory Animal Center of Dalian Medical University (Dalian, China) and injected with lentivirus-p66Shc-shRNA, lentivirus-miR-185-5p-inhibitor, and lentivirus-circ-CBFB-shRNA or lentivirus-scramble (1 × 10^9^ viral particles/mouse) via the tail vein. The lentivirus (systemic-knockdown) was purchased from GenePharma (Shanghai, China). The sense p66Shc lentiviral sequence was 5′-GCTGCATCCCAACGACAAA-3′, the sense miR-185-5p lentiviral sequence was 5′-TCAGGAACTGCCTTTCTCTCCA-3′, and the sense circ-CBFB lentiviral sequence was 5′-AACTGGCTTTTGTGGCTAC-3′. The number of animals in each group was calculated according the formula: *N* = 2 [(a + b)^2^ σ^2^]/(μ_1_ − μ_2_)^2^. The mice were randomly divided into the groups (12 mice/group), with investigators blind to the group allocation. After 7 days, APAP (300 mg/kg, the dose which did not cause death in mice) was injected intraperitoneally into the mice once. The mice were euthanized 24 h after the last injection. All procedures were performed in accordance with the guidelines for the care and use of medical laboratory animals. This study was approved by the Institutional Ethics Committee of Dalian Medical University (Dalian, China). The alanine aminotransferase (ALT) and aspartate aminotransferase (AST) levels were often used to determine the success of APAP liver injury. Therefore, we used the mice (ALT/AST > 200 U/L) for follow-up experiments.

### Pathological detection and biochemical indicators

Paraformaldehyde (4%) was used to fix the liver tissue sections overnight or longer at room temperature, and then the sections were stained with hematoxylin-eosin (H&E). The percentage of necrotic area was quantified by ImageJ software.

The alanine aminotransferase (ALT) and aspartate aminotransferase (AST) levels in the serum were measured by a kit (Jiancheng Corp., Nanjing, China).

The glutathione (GSH), malondialdehyde (MDA), and H_2_O_2_ levels in the mouse liver tissues were detected using kits (Jiancheng Corp., Nanjing, China).

### Cell culture and transfection

The alpha mouse liver 12 (AML12) cell line, which was obtained from the American Type Culture Collection (ATCC, Manassas, VA, USA), was cultured in DMEM: F12 (1:1) containing 10% (v/v) fetal bovine serum as well as insulin (10 μg/ml), transferrin (5.5 μg/ml), selenium (5 ng/ml), and dexamethasone (40 ng/ml). The cells were maintained in a humidified incubator at 5% CO_2_ and 37 °C.

Small interfering RNAs (siRNAs) targeting p66Shc or circ-CBFB, pcDNA-p66Shc plasmid, Ago-185, Ant-185 or negative controls (si-control, pcDNA 3.1, Ago-NC and Ant-NC) were transfected into AML12 cells for 48 h using Lipofectamine 3000 (Invitrogen, Carlsbad, CA, USA). MG132 (Selleck, USA) (10 μm) was added to AML12 cells for 24 h. Mito-TEMPO (Sigma, USA) (100 μM) was added to the AML12 cells for 3 h. Ruboxistaurin (Selleck, USA) (200 nM) was added to the AML12 cells for 48 h. The siRNA sequences targeting p66Shc were sense 5′-GCUGCAUCCCAACGACAAATT-3′, antisense 3′-UUUGUCGUUGGGAUGCAGCTT-5′. The ago-185 sequences were sense 5′-UGGAGAGAAAGGCAGUUCCUGA-3′, antisense 3′-AGGAACUGCCUUUCUCUCCAUU-5′. The ant-185 sequence was sense 5′-UCAGGAACUGCCUUUCUCUCCA-3′. The siRNA sequences targeting circ-CBFB were sense 5′-GGCAGUAACUGGCUUUUGUTT-3′, antisense 3′-ACAAAAGCCAGUUACUGCCTT-5′ and sense 5′-AACUGGCUUUUGUGGCUACTT-3′, antisense 3′-GUAGCCACAAAAGCCAGUUTT-5′. AML12 cells were then treated with APAP (5 mM) for 24 h.

### Western blotting

Liver tissues or AML12 cells were lysed, and protein concentrations were measured. The relative protein expression in liver tissues or AML12 cells was detected by Western blotting. The primary antibodies included antibodies against the following: p66Shc (BD Biosciences, USA, 610878), p-p66Shc (Bioss, China, bs-3410R), CYP2E1 (Proteintech, China, 19937-1-AP), OMA1 (Santa, USA, sc-515788), OPA1 (Proteintech, China, 27733-1-AP), MFN2 (Bioss, China, bs-23685R), DRP1 (Wanleibio, China, WL03028), p-DRP1 (Abcam, USA, ab193216), and beta-actin (Proteintech, China, 60008-1-Ig). Secondary antibodies were obtained from Proteintech. The protein bands were visualized with an enhanced chemiluminescence reagent (ECL, Beyotime, Hangzhou, China).

### RNA isolation and qRT-PCR

TRIzol Reagent (Invitrogen, Carlsbad, CA, USA) was used to extract total RNA. PrimeScript™ RT Reagent Kit (TaKaRa, Japan) was used to synthesize cDNA. SYBR Premix EX Taq™ ii (TaKaRa) was used to perform real-time PCR and specific primers as follows. mmu-miR-185-5p-FO: TCCGCTGGAGAGAAAGGC, mmu-miR-185-5p-RE: ATGGAGGCTGAGGAGCACTG; mmu-circ-CBFB-FO: CGGGAGGAAATGGAGGTG, mmu-circ-CBFB-RE: GGCTAGGTGTTTGTCGCTGTT; mmu-p66Shc-FO: AAGAAGAGCCCCCTGACCAT, mmu-p66Shc-RE: AGGCAGTGTAGCTCCCAAGTG; mmu-OMA1-FO: GAGAGAGACCCCCGCTACCT, mmu-OMA1-RE: TTGTCCATTTGGGAGCACAA; mmu-OPA1-FO: AGATAAGCAACAGTGGGATGCA, mmu-OPA1-RE: CACTGCTCTTGGGTCCGATT; mmu-MFN2-FO: GACCTCCATGGGCATTCTTG, mmu-MFN2-RE: CGCTTGAAGGCCCTCTCTTT; and mmu-DRP1-FO: TTTTCTCGCCCAACGTTGTC, mmu-DRP1-RE: CGGCGAGGATAATGGAATTG. The PCR results, recorded as cycle threshold (Ct) values, were normalized to an internal control (beta-actin or U6).

### Dual immunofluorescence

Paraformaldehyde (4%) was used to fix AML12 cells, and then the primary antibody was incubated with the cells at 4 °C overnight. The next day, cells were incubated with the appropriate secondary antibody (Proteintech) for 1 h at 37 °C. Subsequently, we used DAPI (Beyotime Institute of Biotechnology, Hangzhou, China) to stain the nuclei. The stained cells were observed using a fluorescence microscope.

### MitoSOX staining

Mitochondrial ROS production was detected using MitoSOX Red (Invitrogen). Cells were incubated with MitoSOX for 10 min at 37 °C. Nuclei were stained with Hoechst 33342 (Beyotime Institute of Biotechnology, Hangzhou, China) for 5 min. The immunofluorescence images were obtained with an 80i Nikon microscope (Tokyo, Japan).

### TUNEL staining

TUNEL staining was performed using an apoptosis assay kit (KeyGEN BioTECH, China, KGA704) according to the manufacturer’s instructions.

### Transmission electron microscopy

Mitochondrial morphology was observed using transmission electron microscopy (TEM). AML12 cells were fixed and embedded. Ultrathin sections (50 μm thick) were prepared. Then, the cross section was dehydrated by an ethanol gradient and embedded in epoxy resin. Then, we used citrate and uranyl acetate to stain. A transmission electron microscope (JEOL, Peabody, MA) was used to obtain the images.

### Mitochondria isolation

A mammalian mitochondrial isolation kit (TransGen Biotech, China) was used for mitochondria isolation. The samples were homogenized in Mitochondrial Isolation Buffer using a precooled glass homogenizer. The samples were stored on ice and then centrifuged at 4 °C. The mitochondria were resuspended in Storage Buffer.

### RNA immunoprecipitation and pull-down assays

RNA immunoprecipitation (RIP) assays were detected using an RNA-Binding Protein Immunoprecipitation Kit (Millipore, Bedford, MA, USA). RIP lysis buffer was used to lyse AML12 cells. Then, the whole-cell extract was incubated with RIP buffer containing A + G magnetic beads conjugated with an anti-Argonaute 2 antibody (Ago2, Abcam, ab32381) and normal mouse IgG (Millipore) as a negative control. Then, proteinase K was used to incubate the samples, and the RNA was isolated and determined by qRT-PCR.

Biotin-labeled miR-185-5p mimic, miR-185-5p mut or negative control (GenePharma) was used to transfect AML12 cells using Lipofectamine 3000. M-280 streptavidin magnetic beads (Invitrogen) was used to incubate cell lysates. TRIzol Reagent was used to purify the bound RNAs for further qRT-PCR analysis.

### RNase R treatment

TRIzol was used to extract RNA from AML12 cells. RNase R (GENESEED, Guangzhou, China) was incubated with the total RNA for 15 min at 37 °C. Then, the RNA expression levels of circ-CBFB and CBFB were detected using qRT-PCR.

### Nuclear-cytoplasmic fractionation and fluorescence in situ hybridization

A PARIS Kit (Invitrogen) was used to perform cytoplasmic and nuclear RNA isolation. Then, the RNA expression levels of circ-CBFB, as well as beta-actin (a cytoplasmic protein marker) and U6 (a nuclear protein marker), were examined in the two fractions.

An RNA FISH Kit (GenePharma) was used to detect the distribution of circ-CBFB and miR-185-5p. Hybridization was performed overnight with circ-CBFB and miR-185-5p probes (GenePharma). Nuclei were stained with DAPI. A Nikon inverted fluorescence microscope was used to analyze the specimens.

### Luciferase reporter assay

Luc-p66Shc-mut or luc-CBFB-mut fragments were inserted into a luciferase vector. Then, AML12 cells were transfected with plasmid vectors and Ago-185. A Dual-luciferase Reporter Assay Kit (TransGene Biotech, Beijing, China) was used to measure the luciferase activity.

### Statistical analysis

The data represent the mean ± standard deviation (SD). Data with normal distributions were compared using one-way analysis of variance followed by the Student–Newman–Keuls test. The survival study results were analyzed using the Kaplan–Meier method. Student’s *t* test (two-group comparison) or one-way analysis of variance (more than two groups) was performed for statistical analyses. *P* values <0.05 were considered statistically significant.

## Results

### p66Shc silencing protects mice from APAP-induced mitochondrial dynamics perturbation and liver injury

**Fig. 1:**
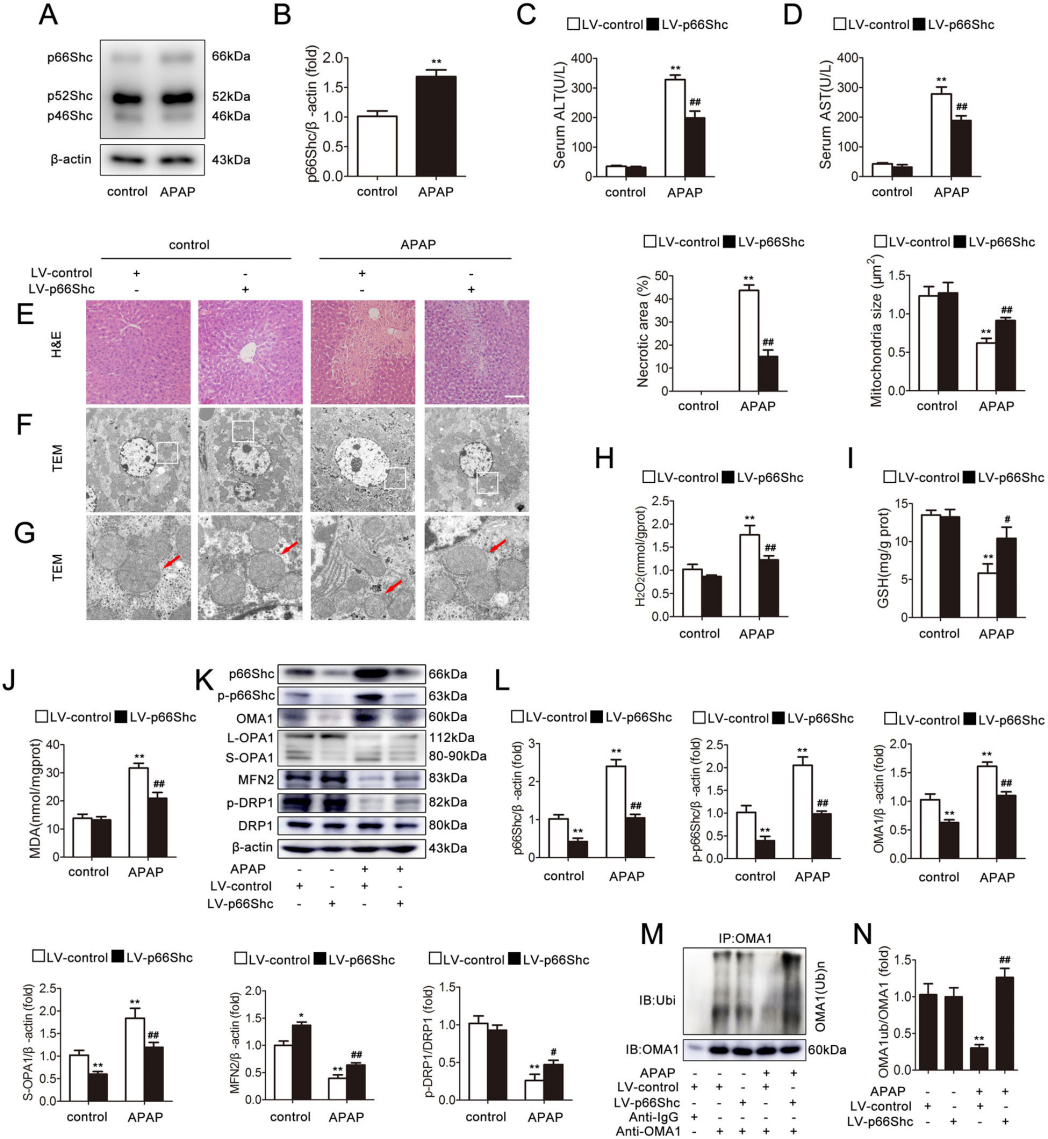
p66Shc silencing protects mice from APAP-induced mitochondrial dynamics perturbation and liver injury. p66Shc silencing was induced via lentiviruses delivered to C57BL/6 mice exposed to APAP (300 mg/kg). **a**, **b** Liver p66Shc, p52Shc, and p46Shc protein, *n* = 3. ^**^p < 0.01 vs. the control group. **c** Serum ALT levels, *n* = 8. **d** Serum AST levels, *n* = 8. **e** H&E staining. Scale bar, 200 μm. **f**, **g** Mitochondrial dynamics perturbation was determined via TEM (×2000/×10,000, magnification, red arrow). **h** Liver H_2_O_2_ levels, *n* = 8. **i** Liver GSH levels, *n* = 8. **j** Liver MDA levels, *n* = 8. **k**, **l** Liver p66Shc, p-p66Shc, OMA1, L-OPA1, S-OPA1, MFN2, DRP1, p-DRP1 protein, *n* = 3. **m**, **n** Liver OMA1 ubiquitination level, *n* = 3. ^*^p < 0.05, ^**^p < 0.01 vs. the LV-control group; ^#^*p* < 0.05, ^##^*p* < 0.01 vs. the APAP group.

Western blotting analysis indicated that p66Shc expression was strongly increased in the liver induced by APAP in comparison to the control both in homogenate and in the mitochondria (Figs. 1a, b and S3A, B). To study the functional contribution of p66Shc to the development of APAP-induced liver injury, p66Shc was silenced via p66Shc-specific lentiviral transduction and was delivered into the mice. Western blotting showed that p66Shc, as well as phospho-p66Shc, was efficiently knocked down in vivo (Fig. 1k). According to H&E staining, the characteristic centrilobular necrosis after APAP overdose is evident in mice, while treatment with p66Shc silencing resulted in significantly less cellular necrosis (Fig. 1e). TEM pictures showed that the majority of mitochondria were scattered and fragmented, exhibiting short rods or sphere shapes in mice treated with APAP. However, p66Shc silencing significantly inhibited the fragmentation of mitochondria (Fig. 1f, g). Consistently, the serum ALT and AST levels, as well as the H_2_O_2_, GSH and MDA activities, revealed that p66Shc knockdown alleviated liver injury (Fig. 1c, d, h–j). Interestingly, the protective effects of lentiviral/siRNA-mediated p66Shc kd on APAP-induced liver injury changed the protein levels of mitochondrial fusion/fission mediators, as evidenced by decreasing OMA1 and S-OPA1 expression, and increasing MFN2 and p-DRP1 expression (Fig. 1k, l). The mRNA level of OMA1, OPA1, MFN2, and DRP1 were also detected in Fig. S1A–E. We found that the OPA1 mRNA level decreased, the MFN2 mRNA level increased, and the OMA1 and DRP1 mRNA levels did not change. These results demonstrated that knockdown of p66Shc attenuates mitochondrial dynamics and liver injury in vivo.

### p66Shc regulates APAP-induced mitochondrial dynamics perturbation in hepatocytes

**Fig. 2:**
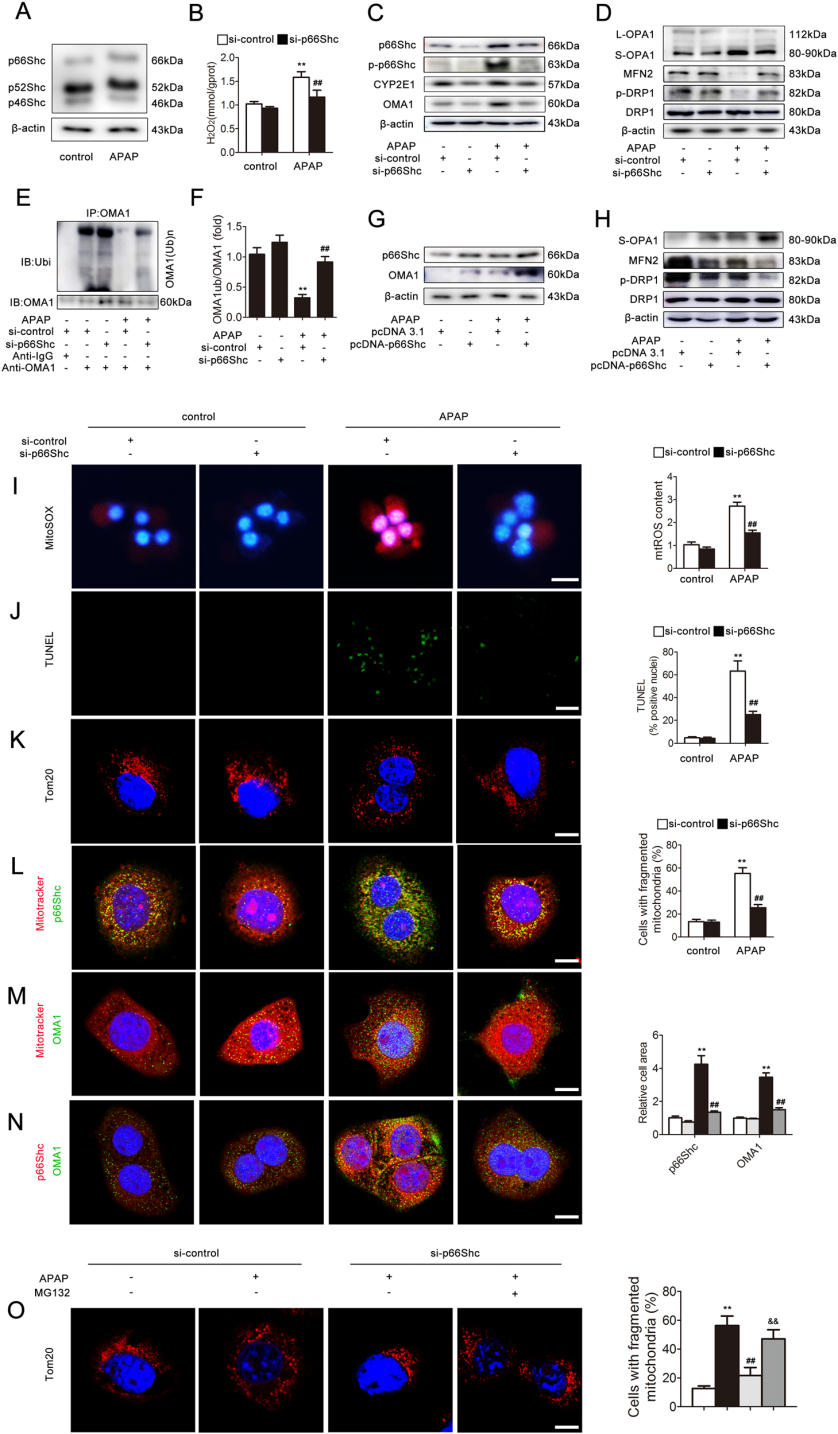
p66Shc regulates APAP-induced mitochondrial dynamics perturbation in hepatocytes. **a** AML12 cells were exposed to APAP (5 mM) and the p66Shc, p52Shc, and p46Shc proteins, *n* = 3. **b**–**f** AML12 cells were transfected with si-control or si-p66Shc and then exposed to APAP (5 mM). **b** H_2_O_2_ levels, *n* = 8. **c**, **d** p66Shc, p-p66Shc, CYP2E1, OMA1, L-OPA1, S-OPA1, MFN2, DRP1, and p-DRP1 proteins, *n* = 3. **e**, **f** OMA1 ubiquitination level, *n* = 3. **g**, **h** AML12 cells were transfected with pcDNA 3.1 or pcDNA-p66Shc and then exposed to APAP and the p66Shc, OMA1, S-OPA1, MFN2, DRP1, and p-DRP1 proteins, *n* = 3. Mitochondrial ROS, cell apoptosis, and mitochondrial fragmentation were determined via representative fluorescence images of MitoSOX- (**i**), Tunel-(**j**), and TOM20-stained (**k**) cells. Scale bar, 200 μm, 100 μm, or 12.5 μm. The colocalization of p66Shc and mitochondria (Mitotracker) (**l**), OMA1 and mitochondria (Mitotracker) (**m**) and p66Shc and OMA1 (**n**). Scale bar, 12.5 μm. (**o**) AML12 cells were transfected with si-control or si-p66Shc and then treated with APAP and MG132. Mitochondrial fragmentation was determined via representative fluorescence images of TOM20-stained cells. Scale bar, 12.5 μm. ^**^p < 0.01 vs. the si-control group; ^##^*p* < 0.01 vs. the APAP group; ^&&^*p* < 0.01 vs. the si-p66Shc group.

Western blotting analysis indicated that p66Shc expression was strongly increased in AML12 cells induced by APAP in comparison to the control in both the homogenate and mitochondria (Fig. 2a and Fig. S3C, D). To further evaluate whether p66Shc mediates APAP-induced hepatocyte injury through a hepatocyte-autonomous mechanism related to mitochondrial dynamics, AML12 cells were transfected with p66Shc siRNA followed by APAP treatment. As shown in Fig. 2b–d, after transfection with p66Shc siRNA in response to APAP, the phospho-p66Shc, OMA1, S-OPA1, and CYP2E1 protein levels and H_2_O_2_ level decreased, and the MFN2 and p-DRP1 protein levels increased. In contrast, p66Shc overexpression increased the OMA1 and S-OPA1 expression and decreased the MFN2 and p-DRP1 expression (Fig. 2g, h). The levels of mtROS and cell death increased after exposure to APAP and were successfully decreased by p66Shc siRNA (Fig. 2i, j). Additionally, as shown in Fig. 2k, fragmented rod- or sphere-shaped morphological characteristics of mitochondria were readily visualized under APAP treatment, whereas mitochondria exhibited filamentous configurations following p66Shc siRNA, suggesting that p66Shc knockdown alleviates the mitochondrial dynamics related to increased mitochondrial fusion and decreased fission. Furthermore, we used PKCβ-inhibitor (Ruboxistaurin) and then detected the expression of phospho-p66Shc and p66Shc (Fig. S2A, B). The results indicated that PKCβ-inhibitor can revert the translocation of p66Shc.

Recent research has demonstrated that the ubiquitination-dependent degradation of OMA1 is deduced to play an essential role in the enhancement of OPA1^37^. Additionally, mtROS has the potential to directly regulate the ubiquitin and SUMO enzymes^38^. Furthermore, we have found that the mRNA level of OMA1 remains unchanged and is inconsistent with its protein level (Figs. 1k and S1B). Thus, it is possible that the effect of p66Shc on mitochondrial dynamics is associated with OMA1 as well as its ubiquitination modification. As shown in Figs. 1m, n and 2e, f, under APAP, the OMA1 ubiquitination level was dramatically increased in response to p66Shc siRNA both in vivo and in vitro, which suggested that p66Shc may increase the expression of OMA1 through inhibiting its ubiquitination under APAP. Additionally, dual-immunofluorescence staining showed that p66Shc (green) and OMA1 (green) were colocalized with Mitotracker (red) in AML12 cells (Fig. 2l, m), and p66Shc (red) and OMA1 (green) were also colocalized in AML12 cells (Fig. 2n), which indicated that there is a correlation between p66Shc and OMA1. Furthermore, the decrease in mitochondrial fragmentation resulting from transfection with p66Shc siRNA was reversed after treatment with MG132 (Fig. 2o), suggesting that OMA1 ubiquitination is required for the regulation of mitochondrial dynamics by p66Shc. Collectively, these results indicate that knockdown of p66Shc attenuates mitochondrial dynamics and APAP-induced liver injury, which may be partially through inhibiting OMA1 ubiquitination.

### p66Shc triggers APAP-induced mitochondrial dynamics perturbation through the regulation of mitochondrial ROS production

**Fig. 3:**
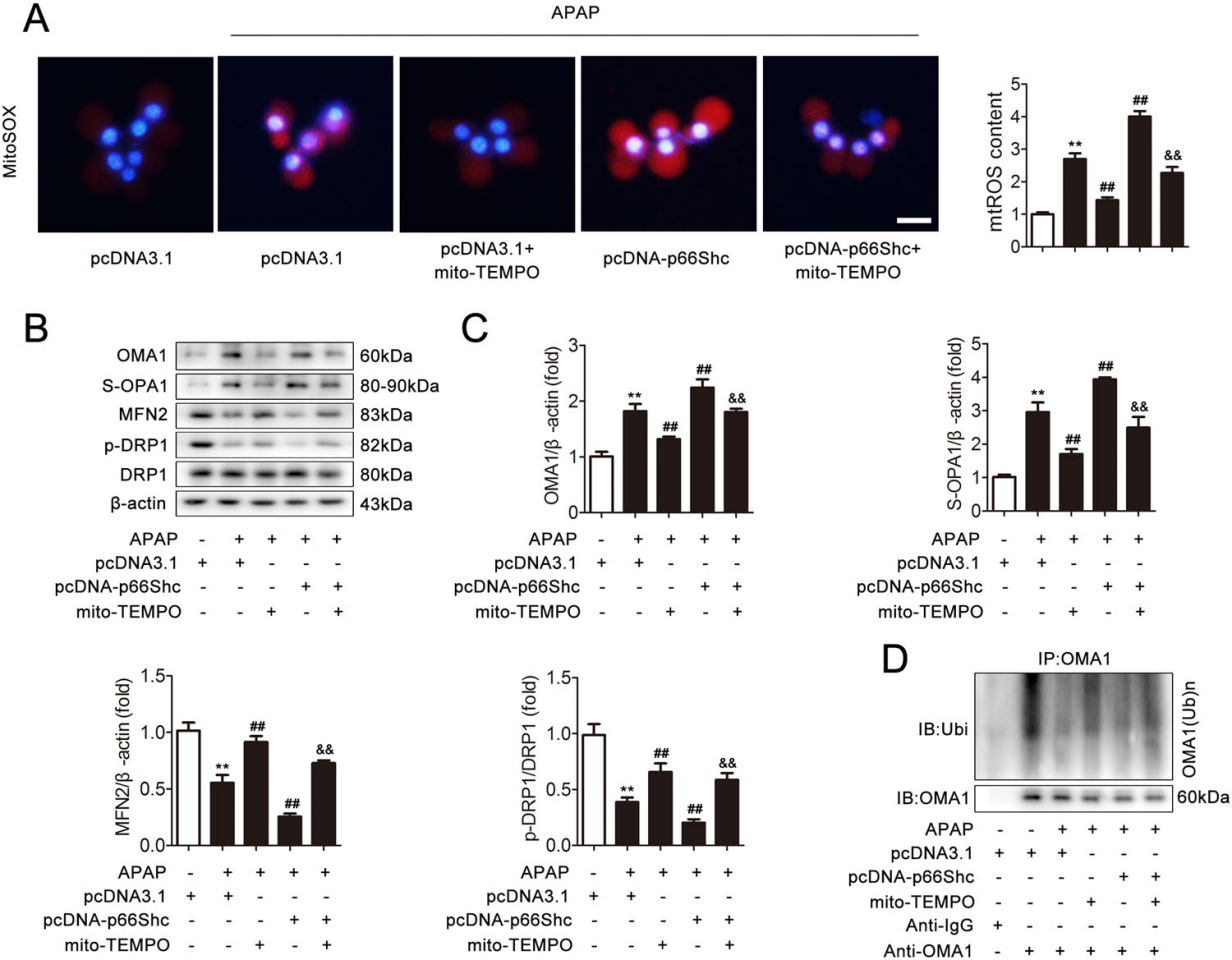
p66Shc triggers APAP-induced mitochondrial dynamics perturbation through the regulation of mitochondrial ROS production. AML12 cells were transected with pcDNA 3.1 or pcDNA-p66Shc and then incubated with mito-TEMPO under APAP treatment. **a** Mitochondrial ROS were determined via representative fluorescence images of MitoSOX-stained cells. Scale bar, 200 μm. **b**, **c** OMA1, S-OPA1, MFN2, DRP1, p-DRP1 protein, *n* = 3. **d** OMA1 ubiquitination level, *n* = 3. ^**^p < 0.01 vs. the pcDNA 3.1 group; ^##^*p* < 0.01 vs. the APAP group; ^&&^*p* < 0.01 vs. the pcDNA-p66Shc group.

To further examine the effect of mitochondrial ROS production mediated by p66Shc on APAP-induced mitochondrial dynamics perturbation, AML12 cells were transfected with pcDNA-p66Shc in the absence or presence of mito-TEMPO, which is a specific mitochondrial ROS scavenger. Under APAP stimulation, p66Shc overexpression resulted in robust ROS production, which was blocked by mito-TEMPO, as detected by MitoSOX (Fig. 3A). Consequently, as shown in Fig. 3b, c, the perturbation of mitochondrial dynamics induced by p66Shc overexpression was significantly abrogated by mito-TEMPO. Furthermore, the reduction of OMA1 ubiquitination induced by p66Shc overexpression was also abrogated by mito-TEMPO (Figs. 3d and S4). Taken together, these data suggest that the underlying mechanism of the effect of p66Shc on APAP-induced mitochondrial dynamics is likely mediated through mitochondrial ROS.

### miR-185-5p participates in the regulation of p66Shc expression

To explore the underlying mechanism by which p66Shc was upregulated upon APAP, we detected whether miRNA could control p66Shc expression. We referred to the miRNA array data from GEO nos. GSE42181 and GSE42182 and a reference^39^ to determine the downregulated miRNAs. We found that 20 miRNAs were decreased (fold change ≥2, *p* < 0.05) in the APAP group compared with those in the control group. Then, the miRNA prediction programs TargetScan and starBase v2.0 were used to determine that miR-185-5p, one of the 20 downregulated miRNAs, targets p66Shc.

**Fig. 4:**
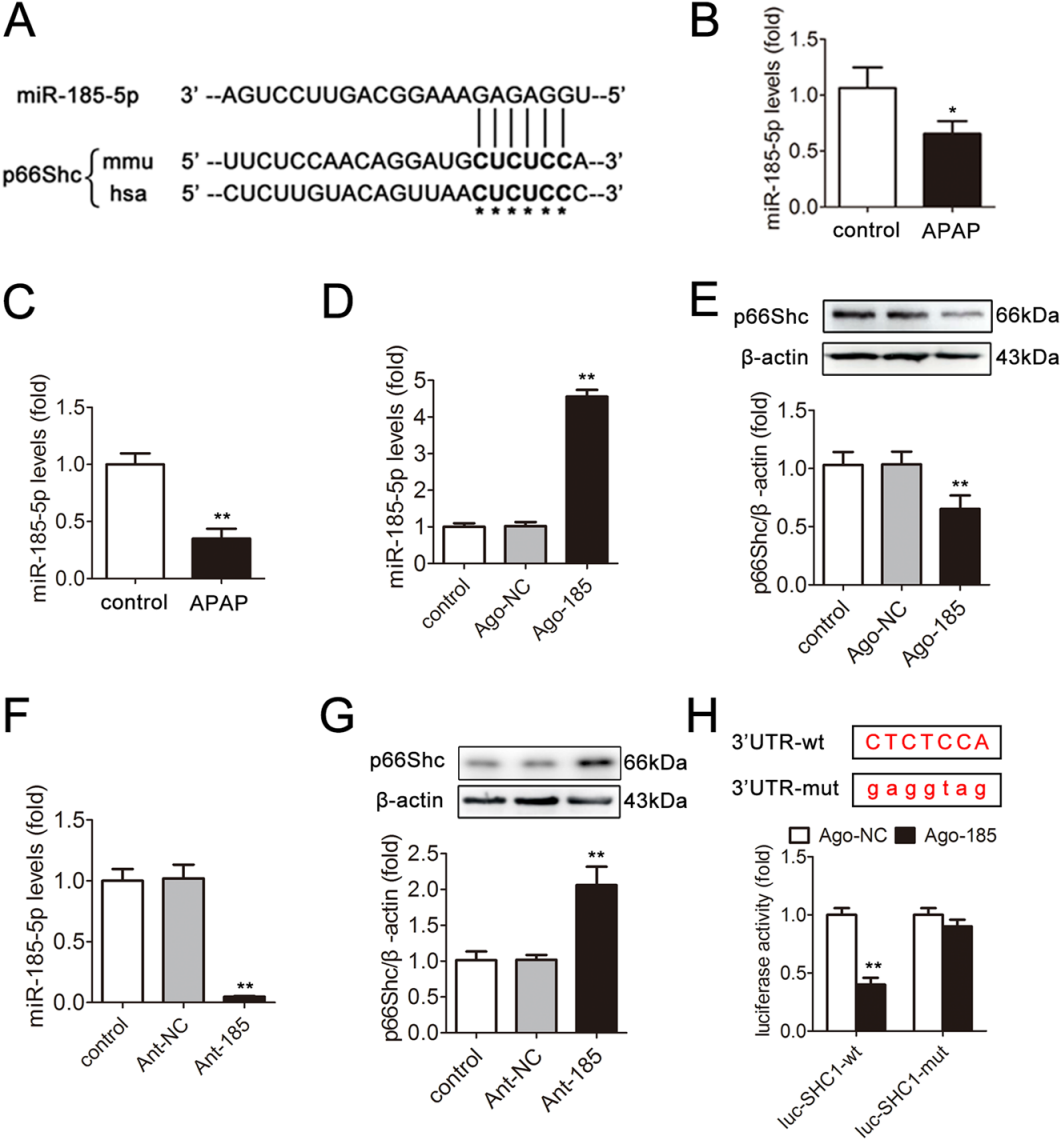
miR-185-5p participates in the regulation of p66Shc expression. **a** miR-185-5p has a predicted binding site in p66Shc from humans and mice. **b** Liver miR-185-5p expression, *n* = 6. **c** Hepatocytes miR-185-5p expression, *n* = 6. ^*^p < 0.05, ^**^p < 0.01 vs. the control group. **d**, **e** AML12 cells were transfected with agomir-NC or miR-185-5p agomir. **d** miR-185-5p expression, *n* = 6. **e** p66Shc protein, *n* = 3. ^**^p < 0.01 vs. the ago-NC group. **f**, **g** AML12 cells were transfected with antagomir-NC or miR-185-5p antagomir. **f** miR-185-5p expression, *n* = 6. **g** p66Shc protein, *n* = 3. ^**^p < 0.01 vs. the ant-NC group. **h** AML12 cells were transfected with wild-type or mutant SHC1-3′-UTR luciferase constructs and with agomir-NC or miR-185-5p agomir. Luciferase activity was detected 48 h after transfection. ^**^p < 0.01 vs. the ago-NC group.

According to bioinformatics analysis, the miR-185-5p binding sequences of p66Shc in the 3′-UTR are highly conserved among different species, including humans and mice (Fig. 4a). The qRT-PCR results showed that the miR-185-5p level was significantly decreased in vivo and in vitro (Fig. 4b, c). We then explored whether miR-185-5p modulation could regulate endogenous p66Shc expression. As shown in Fig. 4d–g, miR-185-5p agomir (ago-185) downregulated the p66Shc protein level, while miR-185-5p antagomir (ant-185) upregulated the p66Shc protein level. Furthermore, a dual-luciferase assay system was used to confirm that miR-185-5p targets the 3′-UTR of p66Shc. As shown in Fig. 4h, after ago-185 transfection, the WT p66Shc 3′-UTR exhibited low luciferase activity. Nevertheless, the mutated p66Shc 3′-UTR abolished the inhibitory effect of ago-185. These data suggest that miR-185-5p targets p66Shc and downregulates its expression in the APAP liver model.

### miR-185-5p attenuates mitochondrial dynamics and APAP-induced hepatocyte injury

**Fig. 5:**
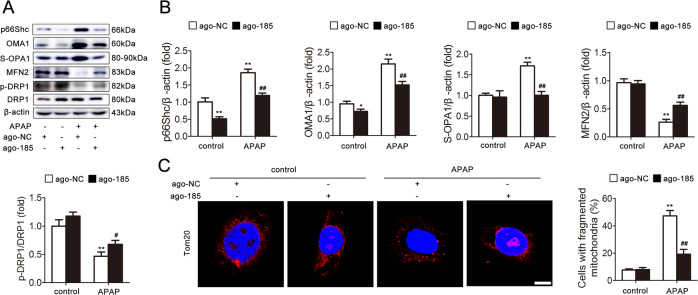
miR-185-5p attenuates mitochondrial dynamics and APAP-induced hepatocyte injury. AML12 cells were transfected with agomir-NC or miR-185-5p agomir and then exposed to APAP. **a**, **b** p66Shc, OMA1, S-OPA1, MFN2, DRP1, and p-DRP1 proteins, *n* = 3. **c** Mitochondrial fragmentation was determined via representative fluorescence images of TOM20-stained cells. Scale bar, 12.5 μm. ^*^p < 0.05, ^**^p < 0.01 vs. the ago-NC group; ^#^*p* < 0.05, ^##^*p* < 0.01 vs. the APAP group.

Subsequently, we investigated the functional role of miR-185-5p in APAP-induced hepatocyte injury. As shown in Fig. 5a, b, enforcing the expression of miR-185-5p through agomir transfection efficiently attenuated the mitochondrial dynamics, as evidenced by the decreasing p66Shc, p-p66Shc, OMA1, and S-OPA1 expression and increasing MFN2 and p-DRP1 expression. Consistently, immunofluorescence staining showed that ago-185 transfection alleviated mitochondrial dynamics by increasing mitochondrial fusion and decreasing fission (Fig. 5c). Thus, our results indicate a possible contribution of miR-185-5p to attenuate mitochondrial dynamics and APAP-induced hepatocyte injury.

### Characterization of circ-CBFB and its interaction with miR-185-5p

**Fig. 6:**
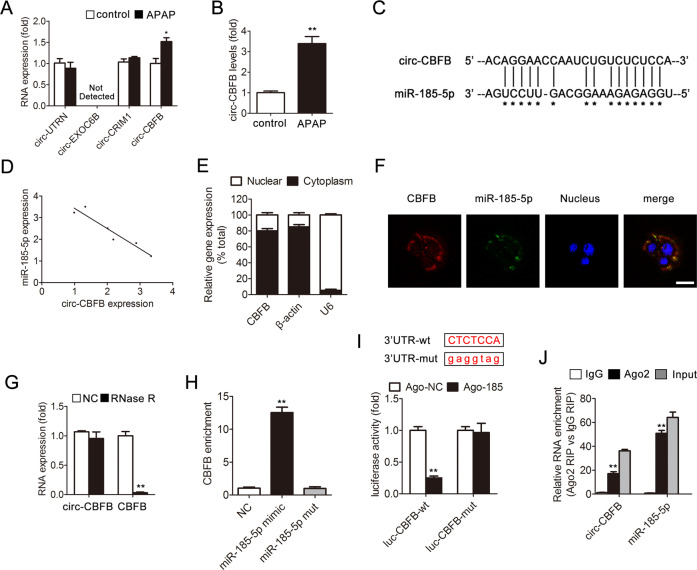
Characterization of circ-CBFB and its interaction with miR-185-5p. **a** Liver circ-UTRN, circ-EXOC6B, circ-CRIM1, circ-CBFB expression, *n* = 6. **b** Hepatocyte circ-CBFB expression, *n* = 6. ^*^p < 0.05, ^**^p < 0.01 vs. the control group. **c** circ-CBFB has a predicted binding site in miR-185-5p. **d** The correlation between circ-CBFB and miR-185-5p expression in liver. **e** Levels of circ-CBFB in the nuclear and cytoplasmic fractions. **f** Dual-immunofluorescence staining of circ-CBFB and miR-185-5p. Scale bar, 50 μm. **g** circ-CBFB and CBFB mRNA expression treated with or without RNase R. ^**^p < 0.01 vs. the NC group. **h** circ-CBFB was pulled down and enriched with a 3′-end biotinylated miR-185-5p mimic. ^**^p < 0.01 vs. the NC group. **i** AML12 cells were transfected with wild-type or mutant circ-CBFB luciferase constructs and with agomir-NC or miR-185-5p agomir. Luciferase activity was detected 48 h after transfection. ^**^p < 0.01 vs. the ago-NC group. **j** AGO2 RIP assay of circ-CBFB and miR-185-5p in AML12 cells. circ-CBFB and miR-185-5p expression was detected by qRT-PCR. ^**^p < 0.01 vs. the IgG group.

CircRNAs have been reported to act as an endogenous RNA sponge to adsorb miRNAs and regulate target gene expression^29–31^. To further understand the underlying mechanism responsible for p66Shc upregulation in response to APAP treatment, we used the circRNA prediction programs CircNet, starBase v2.0, and CircInteractome to identify 19 circRNAs predicted to competitively bind to miR-185-5p. Furthermore, four circRNAs were highly conserved among species. qRT-PCR further confirmed that only circ-CBFB, which is a novel circRNA that derived from the Core-Binding Factor Beta (CBFB) gene locus, was highly expressed in response to APAP treatment (Fig. 6a–c). As expected, the expression of circ-CBFB and miR-185-5p was negatively correlated (Fig. 6d).

To further explore the characterization of circ-CBFB and its interaction with miR-185-5p, qRT-PCR and fluorescence in situ hybridization (FISH) analyses showed that circ-CBFB was abundant and stable in the cytoplasm and colocalized with miR-185-5p (Fig. 6e, f). Resistance to digestion by RNase R (a highly processive 3′–5′ exoribonuclease that digests linear RNAs) indicated that circ-CBFB harbors a secondary loop structure (Fig. 6g). Additionally, circ-CBFB was pulled down by a biotinylated wild-type miR-185-5p mimic, but the introduction of mutations that disrupt base pairing between circ-CBFB and miR-185-5p led to the inability of miR-185-5p to pull down circ-CBFB (Fig. 6h). A luciferase assay indicated the direct binding of circ-CBFB to miR-185-5p (Fig. 6i). Furthermore, RIP of AGO2 in AML12 cells demonstrated that circ-CBFB and miR-185-5p were highly enriched in the AGO2 immunoprecipitation pellet (Fig. 6j). Taken together, these observations suggested that circ-CBFB acts as a miRNA sponge for miR-185-5p in APAP-induced liver injury.

### circ-CBFB functions in APAP-induced hepatocyte injury by targeting miR-185-5p

**Fig. 7:**
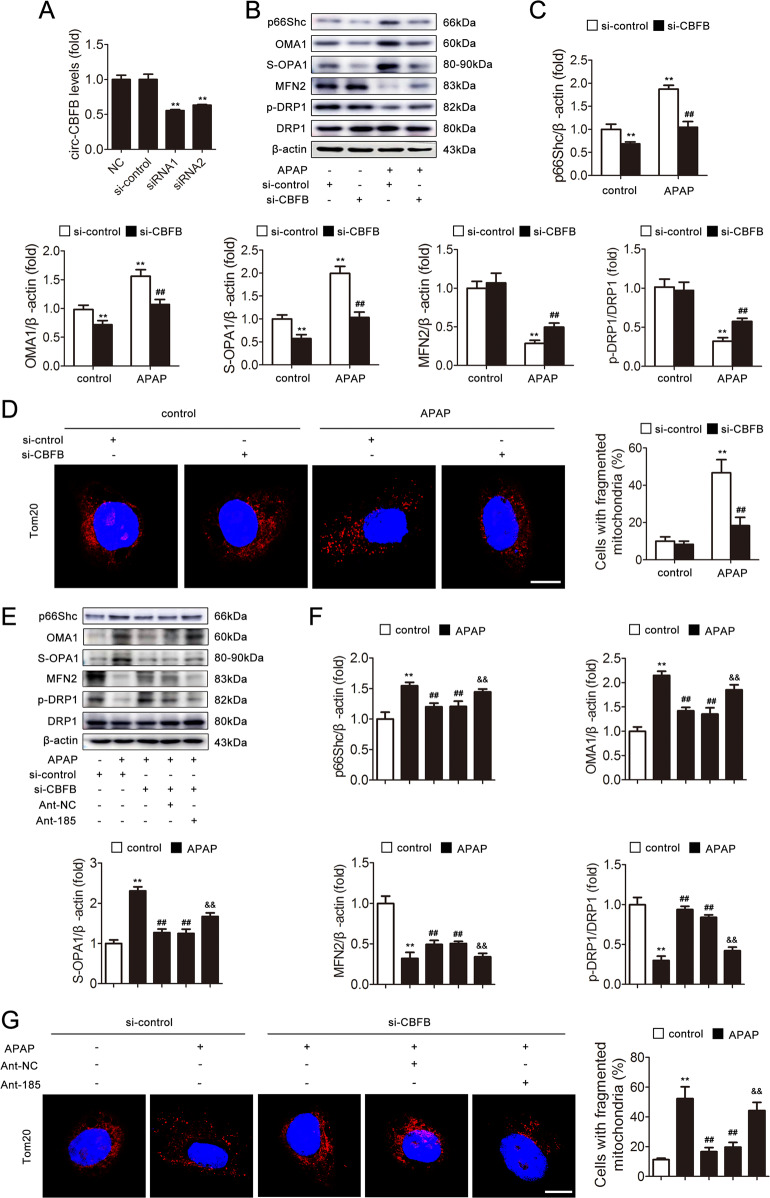
circ-CBFB functions in APAP-induced hepatocyte injury by targeting miR-185-5p. AML12 cells were transfected with si-control or si-CBFB and ant-NC or miR-185-5p antagomir and then exposed to APAP. **a** circ-CBFB expression, *n* = 6. **b**, **c** p66Shc, OMA1, S-OPA1, MFN2, DRP1, and p-DRP1 proteins, *n* = 3. **d** Mitochondrial fragmentation was determined via representative fluorescence images of TOM20-stained cells. Scale bar, 12.5 μm. **e**, **f** p66Shc, OMA1, S-OPA1, MFN2, DRP1, and p-DRP1 proteins, *n* = 3. **g** Mitochondrial fragmentation was determined via representative fluorescence images of TOM20-stained cells. Scale bar, 12.5 μm. ^**^p < 0.01 vs. the si-control group; ^##^*p* < 0.01 vs. the APAP group; ^&&^*p* < 0.01 vs. the si-CBFB group.

To explore whether circ-CBFB functions in APAP-induced hepatocyte injury by interacting with miR-185-5p, we transfected AML12 cells with circ-CBFB siRNA and ant-185 (Fig. 7a). As shown in Fig. 7b–d, the knockdown of circ-CBFB attenuated the hepatocyte injury and perturbation of mitochondrial dynamics induced by APAP. Nevertheless, ant-185 antagonized the protection of circ-CBFB knockdown in APAP-induced hepatocyte injury (Fig. 7e–g). The results indicate that circ-CBFB functions by targeting miR-185-5p in hepatocytes.

### circ-CBFB knockdown attenuates APAP-induced mitochondrial dynamics perturbation and liver injury in mice via miR-185-5p

**Fig. 8:**
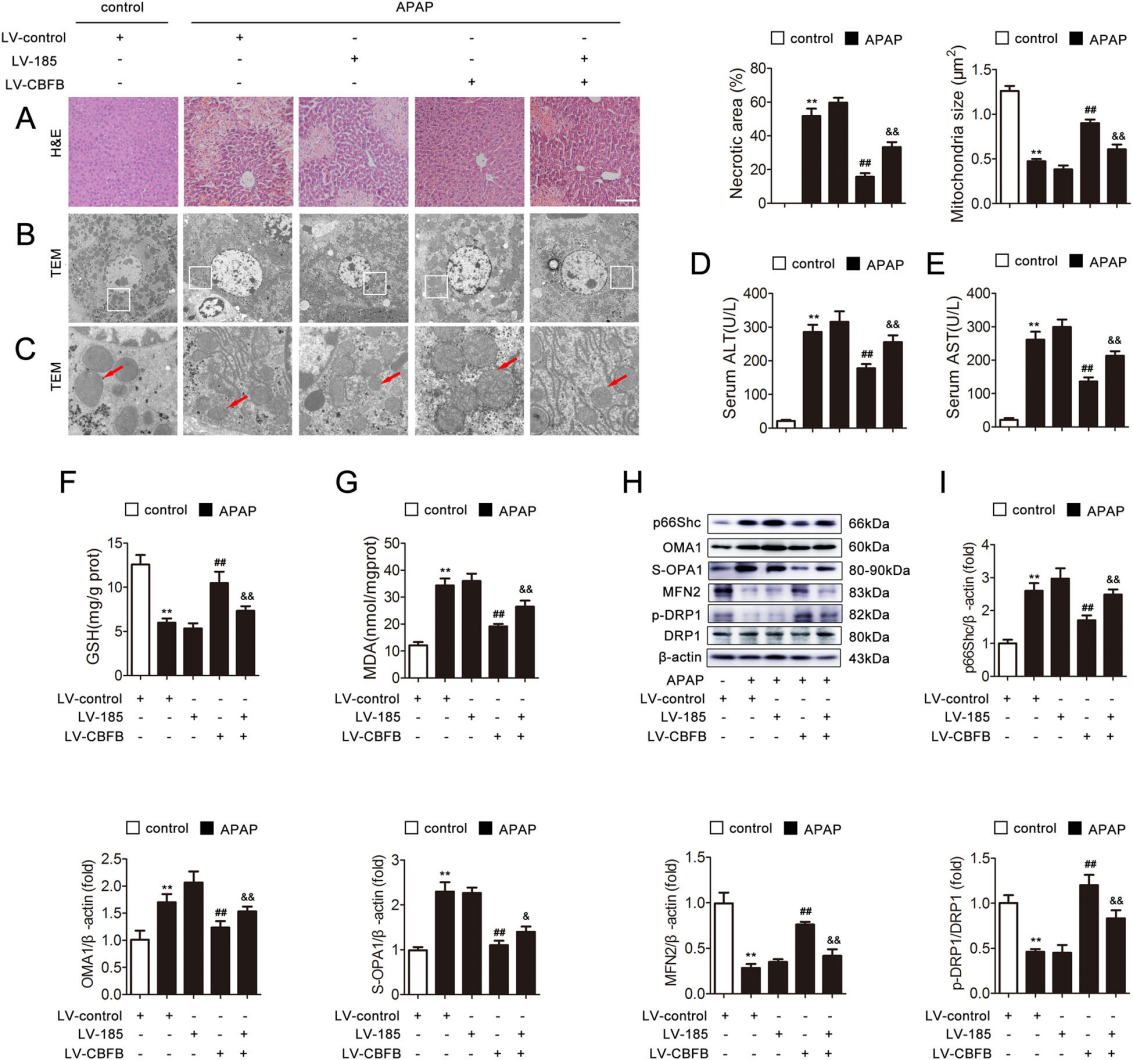
circ-CBFB knockdown attenuates APAP-induced mitochondrial dynamics perturbation and liver injury in mice via miR-185-5p. circ-CBFB and miR-185-5p silencing was induced via lentiviruses delivered to C57BL/6 mice exposed to APAP. **a** H&E staining. Scale bar, 200 μm. **b**, **c** Mitochondrial dynamics perturbation was determined via TEM (×2000/×10,000, magnification, red arrow). **d** Serum ALT levels, *n* = 8. **e** Serum AST levels, *n* = 8. **f** Liver GSH levels, *n* = 8. **g** Liver MDA levels, *n* = 8. **h**, **i** Liver p66Shc, OMA1, S-OPA1, MFN2, DRP1, and p-DRP1 proteins, *n* = 3. ^**^p < 0.01 vs. the LV-control group; ^##^*p* < 0.01 vs. the APAP group; ^&^*p* < 0.05, ^&&^*p* < 0.01 vs. the LV-CBFB group.

To determine the role of circ-CBFB in the regulation of APAP-induced injury, we knocked down circ-CBFB and miR-185-5p expression in mice using lentiviral transduction. As shown in Fig. 8a, the area of cellular necrosis was reduced significantly after circ-CBFB knockdown, while this reduction was markedly rescued by the knockdown of miR-185-5p. In addition, circ-CBFB knockdown substantially rescued the mitochondria with fragmented and disorganized cristae in response to APAP, whereas miR-185-5p knockdown could diminish the effect of circ-CBFB knockdown on mitochondria (Fig. 8b, c). Consistently, circ-CBFB knockdown also alleviated liver injury through miR-185-5p, as evidenced by the serum ALT and AST levels, as well as the GSH and MDA activities (Fig. 8d–g). To further examine the effect of circ-CBFB on mitochondrial dynamics through miR-185-5p, we detected the protein levels of p66Shc, p-p66Shc, OMA1, S-OPA1, MFN2, and p-DRP1. Consequently, the low levels of p66Shc, p-p66Shc, OMA1, and S-OPA1 as well as the high levels of MFN2 and p-DRP1 induced by circ-CBFB knockdown were significantly abrogated by the knockdown of miR-185-5p (Fig. 8h, i). Above all, these data suggest that knockdown of circ-CBFB attenuates liver injury and mitochondrial dynamics in vivo through miR-185-5p.

## Discussion

Drug-induced liver injury is a main cause of liver disease^40^. Indeed, the overuse of APAP is the most common trigger of acute liver failure^1^. Exploring the mechanisms by which APAP causes injury is important to overcome liver damage with appropriate therapies. Here, we first addressed the crucial role of p66Shc in APAP-induced liver injury. We uncovered that p66Shc perturbed mitochondrial dynamics execution in the liver and hepatocytes. Furthermore, we identified circ-CBFB as a ceRNA that mediates p66Shc in APAP-induced liver injury.

Mitochondria are the primary intracellular organelles in response to APAP since mitochondrial bioenergetic inhibition is an early event in APAP-induced liver injury^41^. APAP hepatotoxicity is an intricate process that involves mitochondrial oxidant stress generation, mitochondrial dynamics perturbation, mitochondrial fragmentation, and hepatocyte cell death and regeneration^5^. Our results provide a mode of action of APAP in mitochondrial dysfunction, including oxidative stress generation, dynamic perturbation, and mitochondrial fragmentation. We found that p66Shc, a major regulator of mitochondrial ROS, played an essential role in mediating mitochondrial dynamics perturbation and mitochondrial fragmentation in response to APAP, both in vivo and in vitro. Additionally, p66Shc knockdown increased the ubiquitination level of OMA1, and MG132 abolished the effect of p66Shc on mitochondrial fragmentation, suggesting that the effect of p66Shc on mitochondria was partially exerted by inhibiting OMA1 ubiquitination. Therefore, p66Shc is a crucial mediator of mitochondrial dysfunction in APAP-induced liver injury.

In recent years, gene-targeting therapy has attracted more attention, and exploring novel genes and effective drug targets is key to the individualized treatment of diseases^42,43^. It has been reported that the Hsv-tk/GCV and CD/5-fc systems are the most common target genes in hepatocellular carcinoma treatment^44^. Additionally, the discovery of lung cancer driver genes and their targeted inhibitors, such as epidermal growth factor receptor (EGFR) and ALK receptor tyrosine kinase inhibitors, has achieved remarkable clinical benefits^45,46^. However, the clinical application of gene therapy is currently limited to the field of cancer. Thus, it is particularly important to explore potential therapeutic targets and their clinical application prospects for other diseases. Our previous studies have demonstrated that p66Shc is associated with hepatocyte oxidative stress during chronic alcoholic liver injury and NAFLD^18,19^. Furthermore, we have also shown that silencing p66Shc in vivo significantly alleviated the development of fibrosis and HSC activation^47^. In this study, we discovered that silenced p66Shc protects mice from APAP-induced mitochondrial dysfunction and liver injury. Therefore, it is conceivable that p66Shc can serve as a potential therapeutic target for liver diseases, and gene-targeting therapy for p66Shc may have good application prospects for the treatment of clinical liver diseases.

Circular RNAs are endogenous noncoding RNAs, are conserved in mammalian cells, are tissue-specific and extensively studied, and have great potential that remains to be explored^24^. An increasing number of studies have shown that circRNAs play essential regulatory roles in multiple pathological and physiological processes^48,49^. However, it is not yet clear whether circRNAs are involved in the progression of APAP-induced liver injury, as well as with p66Shc. Therefore, exploring the mechanisms of circRNAs in p66Shc and APAP-induced liver injury may provide a new perspective for the treatment of liver injury. In this study, we first identified circ-CBFB as a key upregulated circRNA involved in APAP-induced liver injury. In addition, we used loss-of-function approaches both in vivo and in vitro to demonstrate the effect of circ-CBFB on p66Shc in response to APAP. We revealed a new function of circ-CBFB in APAP-induced liver injury progression and identified it as a therapeutic target for liver injury treatment.

Gene silencing is an important tool for exploring the progression of diseases. Currently, lentiviral vectors^50^, adenoviral vectors^51^, adeno-associated viruses^52^, and siRNAs^53^ have been widely used to knockdown target genes in vivo. Due to the advantages of a long knockdown time, the ability to integrate into the genome, low immunogenicity, and low cost compared with those characteristics of other technologies, we delivered lentiviral vectors into mice. However, the sequences of lentiviral vectors cannot be amplified, and the immunogenicity of lentiviral vectors is slightly higher than that of adeno-associated viruses. In general, gene silencing is of great significance in the exploration of APAP-induced liver injury. The field is now advancing towards clinically applicable gene editing technologies in which gene silencing plays an important role, and proof-of-concept studies are now being realized^54^.

Our work describes, for the first time, the role of p66Shc in the control of the developmental potential of APAP-induced liver injury by exacerbating mitochondrial dysfunction. Our results further reveal that circ-CBFB acts as a miR-185-5p sponge to regulate APAP-induced liver injury by targeting p66Shc. In summary, our data offer novel therapeutic targets, circ-CBFB and p66Shc, to broaden the treatment options for APAP-induced liver injury.

## Supplementary information

supplementary figure legends

Supplementary Figure 1

Supplementary Figure 2

Supplementary Figure 3

Supplementary Figure 4

Supplementary Figure 5

Supplementary Figure 6

Supplementary Figure 7
