# Supplementary material for: circ-CBFB upregulates p66Shc to perturb mitochondrial dynamics in APAP-induced liver injury
Source: Cell Death Dis. 2020 Nov 6;11(11):953. doi: 10.1038/s41419-020-03160-y (PMC7648761; doi:10.1038/s41419-020-03160-y)
Supplement: Supplementary file 1 — supplementary figure legends [file 41419_2020_3160_MOESM1_ESM.docx]

**Supplementary figure legends**

**Figure S1. The mRNA levels of mitochondrial dynamics-related proteins.** p66Shc silencing was induced via lentiviruses delivered to C57BL/6 mice exposed to APAP (300 mg/kg). (A-E) Liver p66Shc, OMA1, OPA1, MFN2 and DRP1 mRNA levels, n=6. ^**^p < 0.01 vs. the LV-control group; ^##^p < 0.01 vs. the APAP group.

**Figure S2. PKCβ-inhibitor could revert the translocation of p66Shc.** AML12 cells were incubated with Ruboxistaurin and then exposed to APAP. (A, B) p66Shc and p-p66Shc proteins, n=3. ^*^p < 0.05, ^**^p < 0.01 vs. the control group; ^##^p < 0.01 vs. the APAP group.

**Figure S3. The protein levels of mitochondrial p66Shc in vivo and in vitro.** (A, B) Mitochondrial p66Shc protein in mouse liver, n=3. (C, D) Mitochondrial p66Shc protein in AML12 cells, n=3. ^**^p < 0.01 vs. the control group.

**Figure S4. p66Shc inhibits OMA1 ubiquitination through mitochondrial ROS.** (A) AML12 cells were transected with pcDNA 3.1 or pcDNA-p66Shc and then incubated with mito-TEMPO. OMA1 ubiquitination level, n=3. ^**^p < 0.01 vs. the pcDNA 3.1 group; ^##^p < 0.01 vs. the pcDNA-p66Shc group.

**Figure S5. Quantitative results of proteins related to mitochondrial dynamics.** (A) AML12 cells were transfected with si-control or si-p66Shc and then exposed to APAP (5 mM) and the p66Shc, p-p66Shc, CYP2E1, OMA1, S-OPA1, MFN2 and p-DRP1 proteins were quantified, n=3. ^*^p < 0.05, ^**^p < 0.01 vs. the si-control group; ^#^p < 0.05, ^#^^#^p < 0.01 vs. the APAP group. (B) AML12 cells were transfected with pcDNA 3.1 or pcDNA-p66Shc and then exposed to APAP and the p66Shc, OMA1, S-OPA1, MFN2 and p-DRP1 proteins were quantified, n=3. ^**^p < 0.01 vs. the pcDNA 3.1 group; ^##^p < 0.01 vs. the APAP group.

**Figure S6. circ-CBFB upregulates p66Shc to perturb mitochondrial dynamics in APAP-induced liver injury.** circ-CBFB interacts with and upregulates p66Shc, which inhibits OMA1 ubiquitination and induces mitochondrial dynamics perturbation.
