# Supplementary figures and images for: circ-CBFB upregulates p66Shc to perturb mitochondrial dynamics in APAP-induced liver injury
Source: Cell Death Dis. 2020 Nov 6;11(11):953. doi: 10.1038/s41419-020-03160-y (PMC7648761; doi:10.1038/s41419-020-03160-y)

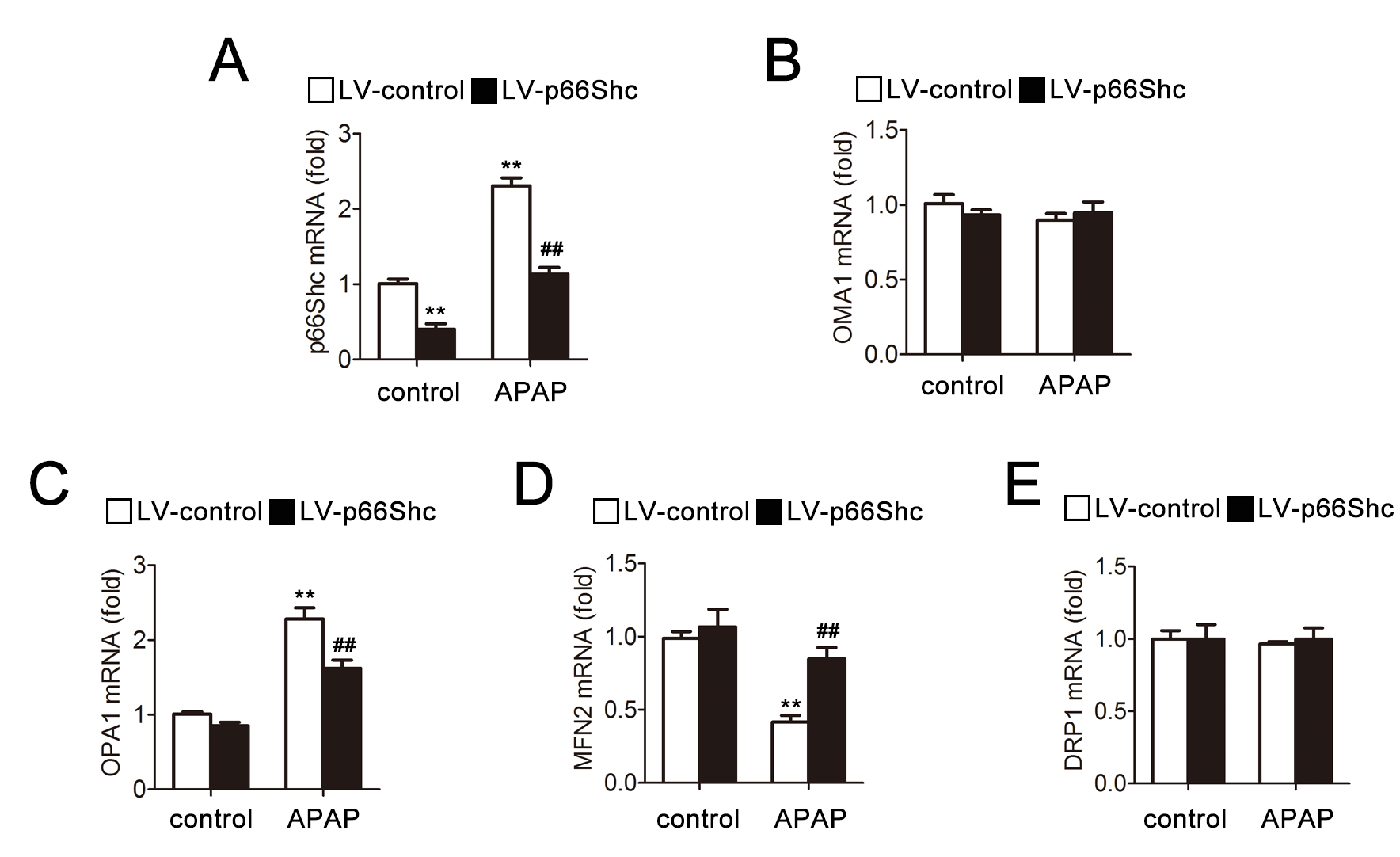

Supplement: Supplementary file 2 — Supplementary Figure 1 [file 41419_2020_3160_MOESM2_ESM.tif]

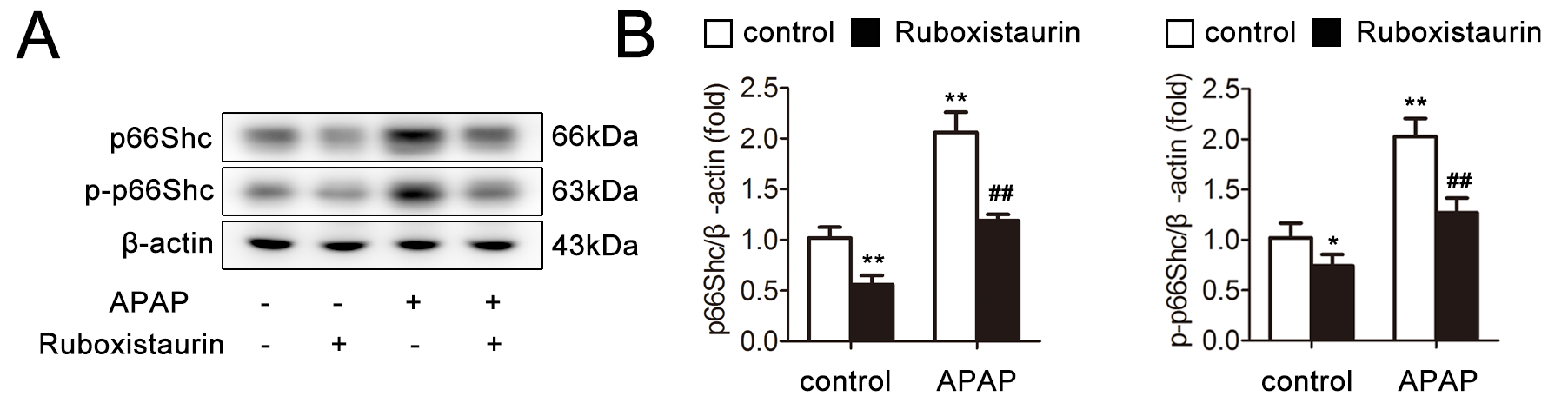

Supplement: Supplementary file 3 — Supplementary Figure 2 [file 41419_2020_3160_MOESM3_ESM.tif]

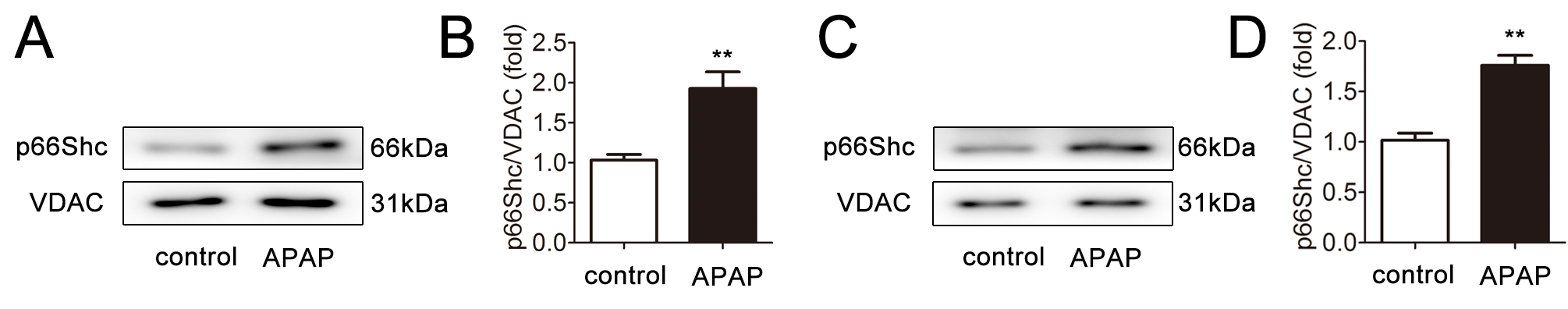

Supplement: Supplementary file 4 — Supplementary Figure 3 [file 41419_2020_3160_MOESM4_ESM.tif]

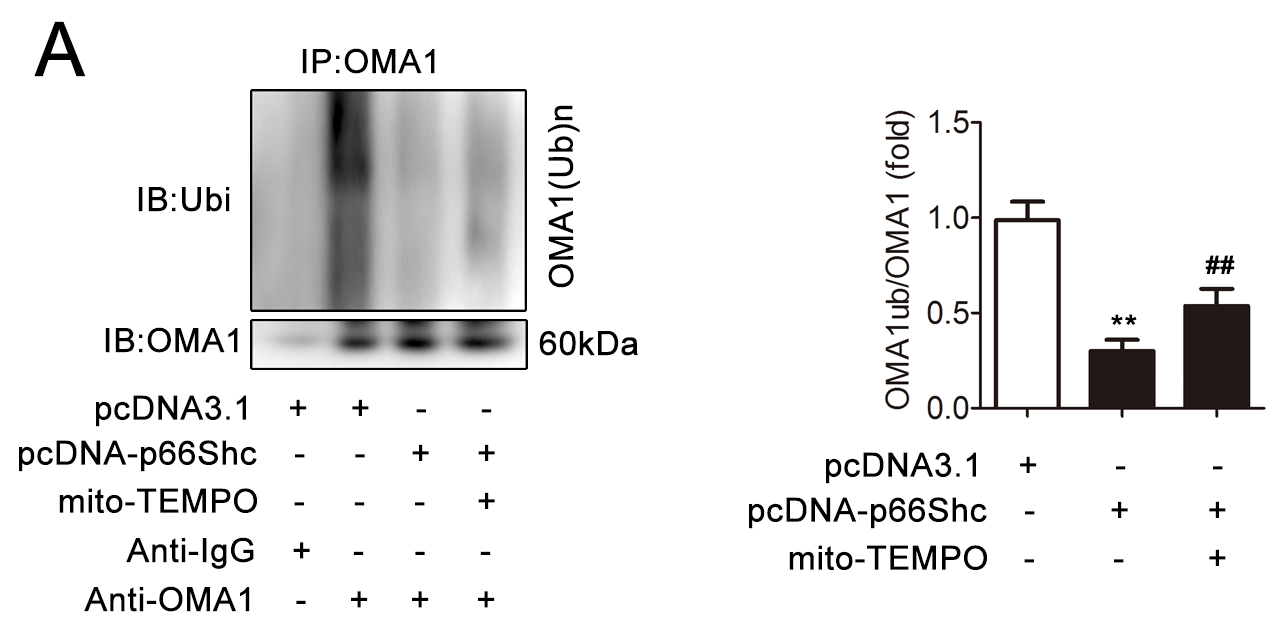

Supplement: Supplementary file 5 — Supplementary Figure 4 [file 41419_2020_3160_MOESM5_ESM.tif]

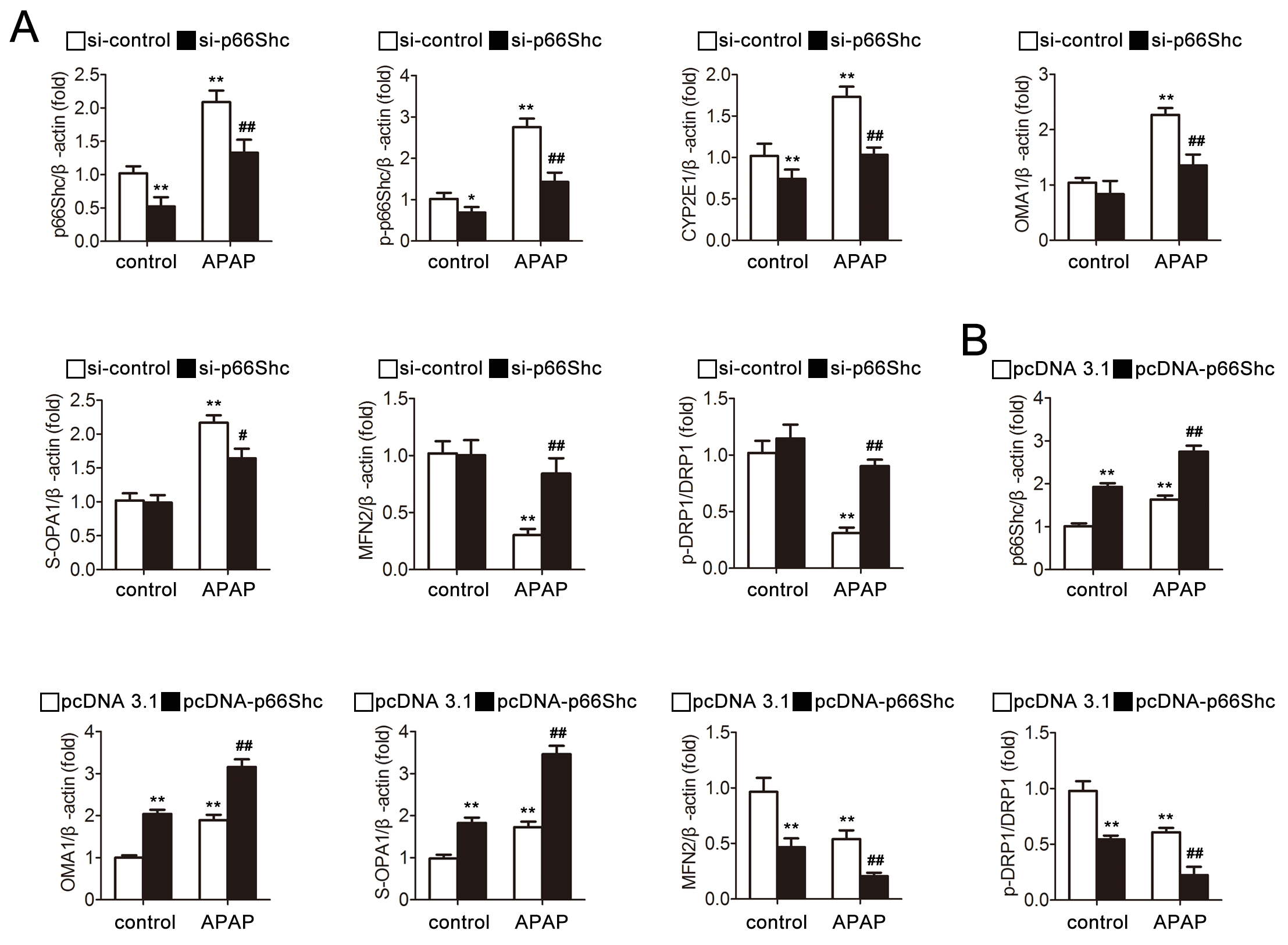

Supplement: Supplementary file 6 — Supplementary Figure 5 [file 41419_2020_3160_MOESM6_ESM.tif]

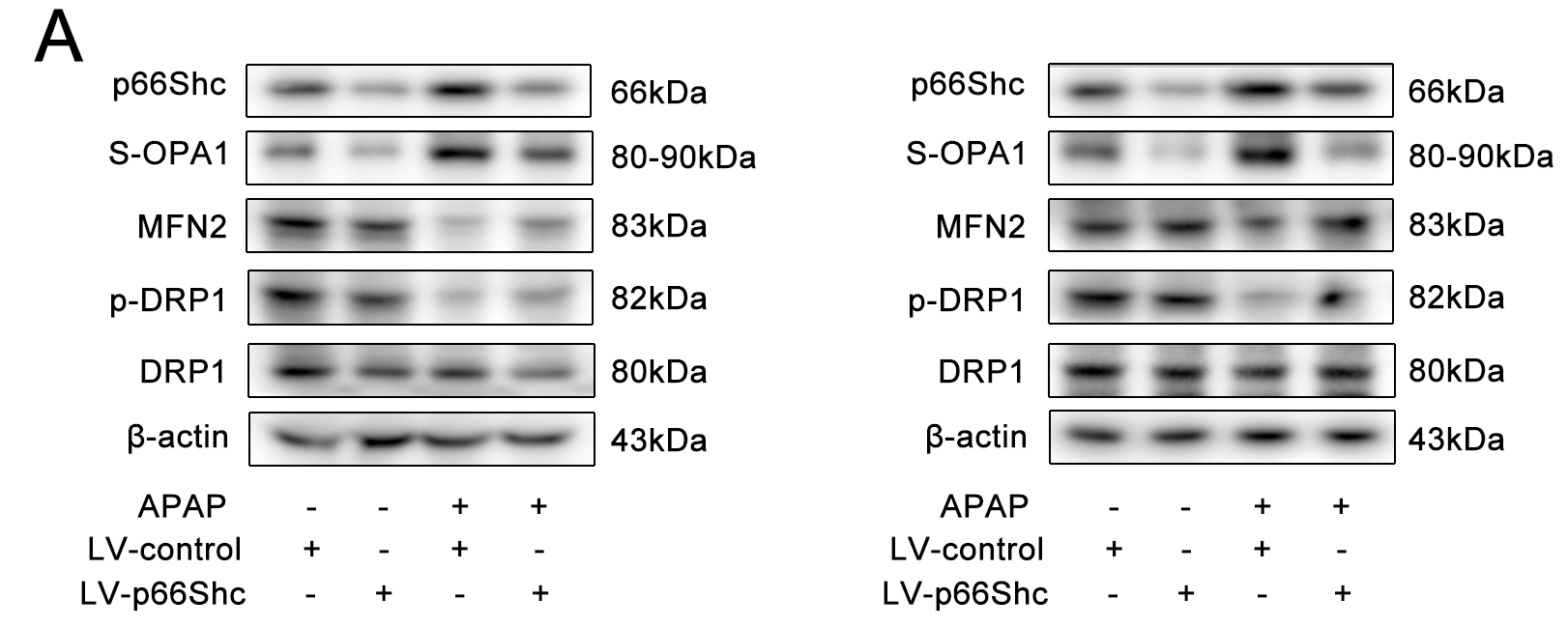

Supplement: Supplementary file 7 — Supplementary Figure 6 [file 41419_2020_3160_MOESM7_ESM.tif]

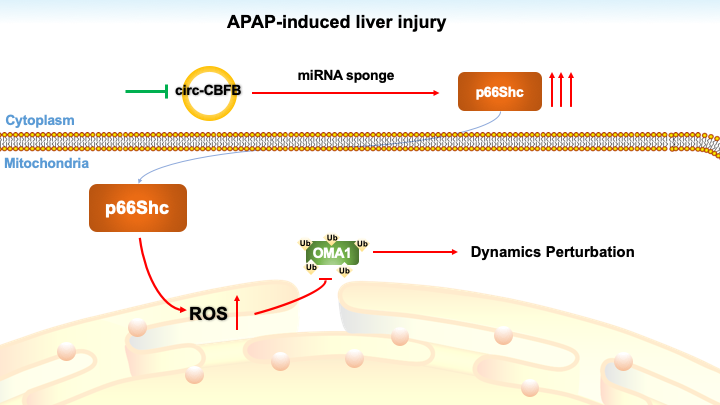

Supplement: Supplementary file 8 — Supplementary Figure 7 [file 41419_2020_3160_MOESM8_ESM.tif]
